# hsa_circ_0000285 sponging miR-582-3p promotes neuroblastoma progression by regulating the Wnt/β-catenin signaling pathway

**DOI:** 10.1515/med-2023-0726

**Published:** 2023-07-14

**Authors:** Jun Du, Yingquan Zhuo, Xu Sun, Meilan Nie, Jiafei Yang, Xi Luo, Huajian Gu

**Affiliations:** Department of Pediatric Surgery, The Affiliated Hospital of Guizhou Medical University, Guiyang, Guizhou 550004, China; Department of Pediatric Surgery, The Affiliated Hospital of Guizhou Medical University, No. 16 Beijing Road, Yunyan District, Guiyang, Guizhou 550004, China

**Keywords:** circ_0000285, neuroblastoma, miR-582-3p, Wnt/β-catenin

## Abstract

Circular RNA has been reported to play a key role in neuroblastoma (NB); however, the role of circ_0000285 in NB remains unclear. The aim of this study was to elucidate the role of circ_0000285 in NB. We studied the expression patterns of miR-582-3p and circ_0000285 in NB tissues and cells using real-time quantitative polymerase chain reaction. The expression of proteins associated with apoptosis (Bax and Bcl-2) and the proteins associated with Wnt/β-catenin (Wnt, p-Gsk-3β, Gsk-3β, β-catenin, and C-myc) were quantified by western blotting. *In vivo* animal models were prepared for the functional verification of circ_0000285 on tumor growth. The potential binding of circ_0000285 to miR-582-3p was ascertained using dual-luciferase reporter and RNA-binding protein immunoprecipitation experiments. Noticeable upregulation of circ_0000285 expression was observed in NB tumor samples and cell lines. *In vivo* and *in vitro* experiments indicated that the absence of circ_0000285 repressed NB cell proliferation and migration, provoked apoptosis, and impaired the activity of Wnt/β-catenin signaling. miR-582-3p is targeted by circ_0000285 and is poorly expressed in NB cells. The additional repression of miR-582-3p in NB cells after circ_0000285 silencing largely recovered circ_0000285 silencing-suppressed NB cell proliferation and migration and enhanced apoptosis. The absence of miR-582-3p restored Wnt/β-catenin signaling activity impaired by the knockdown of circ_0000285. circ_0000285 functions as an miR-582-3p sponge to strengthen Wnt/β-catenin signaling activity, thus exacerbating NB development.

## Graphical abstract

**Figure:**
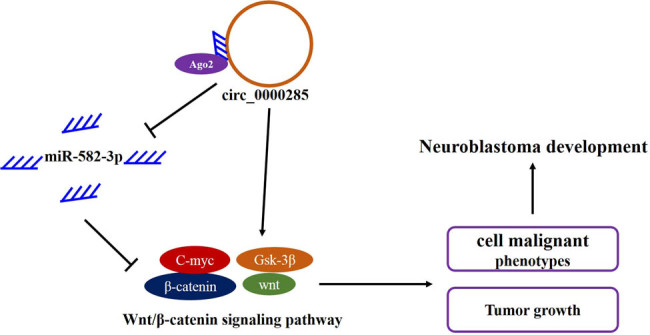

## 1 Introduction

Neuroblastoma (NB), formed by sympathetic nerve cells, is a heterogeneous solid tumor that can occur in any part of the sympathetic nervous system [1,2]. According to a recent report, NB makes up 7–8% of all childhood cancer cases and about 15% of deaths due to childhood cancer [3]. Despite multimodal treatment, most patients with recurrent high-risk NB die from the disease, with a 5-year survival rate of <50% [3,4]. Functional characterization of oncogenes or tumor suppressor genes in NB is needed to provide important insights into the unknown mechanisms of NB development and offer additional therapeutic strategies.

The definition of circular RNA (circRNA) as an oncogenic factor or cancer suppressor based on its function in cancer biology has generated widespread interest [5]. circRNA originates from precursor mRNA via a “back-splicing” mechanism, which is characterized by continuous loop-closed structures. With the wide application of RNA-sequencing technology, numerous circRNAs with tissue- or developmental-stage-specific expression have been identified in diverse cancers [6]. Accumulating evidence indicates that circRNA deregulation is linked to carcinogenesis by mediating various cellular biological functions, such as proliferation, survival, and energy metabolism [7,8]. Moreover, circRNA deregulation is associated with the activation or inactivation of several signaling pathways closely related to cancer development, such as PI3K/AKT and Wnt/β-catenin signaling [9,10]. Yang et al. revealed that circ_0133622 contributes to NB progression by enhancing NB cell growth, survival, migration, and invasion [11]. Given that the vital functionality of circRNAs in biology is newly recognized, there is still a large gap in our understanding of the effects and molecular mechanisms of circRNA action in different cancers. Herein, we focused on circ_0000285, which is derived from the HIPK3 mRNA. Studies have shown that it can be used as a driver of oncogenic development in several cancers, including cervical cancer and osteosarcoma [12,13]. To our knowledge, the function and mechanism of circ_0000285 in NB have rarely been reported.

It is canonical that circRNAs may exert effects by functioning as miRNA sponges. The potential binding of circ_0000285 to downstream miRNAs can be predicted by public bioinformatics tools, such as circInteractome and starBase [14,15]. Here, we obtained results from bioinformatic analysis that miR-582-3p could be targeted by circ_0000285. Previous research has shown that miR-582-3p has conflicting effects on a variety of cancers [16,17]. However, its role in NB remains unclear. The interplay between circ_0000285 and miR-582-3p in cancer development has not been clarified.

The purpose of the present study was to explore the function of circ_0000285 in NB progression using loss-of-function assays. In addition, we addressed the effects of circ_0000285 deregulation on the activation of canonical Wnt/β-catenin signaling and validated the interactions between circ_0000285 and miR-582-3p, thereby proposing a novel mechanism illustrating the role of circ_0000285 in NB.

## 2 Materials and methods

### 2.1 Clinical samples

Patients with NB were first diagnosed via biopsy or surgical pathology and underwent surgery at the Affiliated Hospital of Guizhou Medical University. Tumor samples (*n* = 23) and non-cancerous normal tissues (*n* = 23) from patients who provided written informed consent and did not receive chemotherapy or radiotherapy were used in our study. Patients with severe systemic diseases were excluded from the study.

### 2.2 Cell culture

Several NB cell lines, including SK-N-BE (Catalog No: BNCC337645), SK-N-SH (Catalog No: BNCC359892), SK-N-AS (Catalog No: BNCC342030), and IMR-32 (Catalog No: BNCC359788), and non-cancerous HEK293 (Catalog No: BNCC100449) cells were purchased from BeNa (China). SK-N-BE cells were maintained in F-12K complete medium (Catalog No: BNCC338550; BeNa) containing 10% fetal bovine serum (FBS), and all other cell lines were maintained in Dulbecco’s modification of eagle’s medium complete medium (Catalog No: BNCC363314; BeNa) containing 10% FBS and 5% CO_2_ at 37°C.

### 2.3 Real-time quantitative polymerase chain reaction (RT-qPCR)

A commercial TRIzol reagent (Catalog No: 15596018; Invitrogen, USA) was used to extract the total RNA. For the RNase R assay, the total RNA was treated with 2 U/μg RNase R at 37°C for 20 min. Next, RNA samples were subjected to reverse transcription using the High-Capacity cDNA Reverse Transcription Kit (Catalog No: 4368814; Applied Biosystems, USA) and quantified for RT-qPCR analysis using UltraSYBR Mixture (Catalog No: CW2601M; Cwbio, China). The Quant Studio6 PCR system (Life Technologies, USA) was used for RT-qPCR. For RT-qPCR of miRNAs, cDNA synthesis and quantification were conducted using an miRNA Real-Time PCR Assay Kit (Catalog No: CW2142; Cwbio). All experimental procedures were performed in accordance with the manufacturer’s protocol. In this study, relative expression was identified using the 2^−△△Ct^ method, with GAPDH (for circ_0000285) and U6 (for miR-582-3p) as the housekeeping genes for circRNA and miRNA, respectively. Primer sequences are shown in Table 1.

**Table 1:** Real-time PCR primer synthesis list

Gene	Sequences	
circ_0000285	Forward	5′-TACCTCTGCAGGCAGGAACT-3′
	Reverse	5′-TCACATGAATTTAGGTGGGACTT-3′
miR-582-3p	Forward	5′-GCACCATTGAAGAGGACAGAC-3′
	Reverse	5′-TATTGAAGGGGGTTCTGGTG-3′
U6	Forward	5′-CTCGCTTCGGCAGCACA-3′
	Reverse	5′-AACGCTTCACGAATTTGCGT-3′
GAPDH	Forward	5′-AGAAAAACCTGCCAAATATGATGAC-3′
	Reverse	5′-TGGGTGTCGCTGTTGAAGTC-3′

### 2.4 Subcellular location

For location analysis of circ_0000285, a commercial PARIS Kit (Catalog No: AM1921; Thermo Fisher Scientific, USA) was used to separate RNA samples from the cytoplasmic and nuclear fractions of NB cells. The abundance of circ_0000285 in the separated fractions was determined using RT-qPCR. GAPDH was used as the internal control for the cytoplasm, and U6 was used as the internal control for the nucleus.

### 2.5 Cell transfection

Small-interfering RNA targeting circ_0000285 (si-circ), circ_0000285 overexpression vector (OE-circ), and their negative controls (si-NC and OE-NC) were obtained from Geneseed (China). miR-582-3p mimic (miR-582-3p), mimic NC (miR-NC), miR-582-3p inhibitor (inhibitor), and inhibitor-NC were purchased from Ribobio (China). Cells were transfected with siRNA (40 nM), miRNA mimic (40 nM), overexpression vectors (100 ng), or miRNA inhibitor (60 nM) using Lipofectamine 3000 (Catalog No: L3000015; Invitrogen). Cells collected after 24 h transfection were analyzed by RT-qPCR or western blotting to verify the efficacy of transfection.

### 2.6 Cell counting kit-8 (CCK-8) assay

Cells containing different transfections were seeded on plates of 96 wells (5 × 10^3^ cells/well). Cells were then cultured at 37°C, and 10 μL/well CCK-8 reagent (Catalog No: C0038; Beyotime, China) was used to treat cells at 0, 24, 48, or 72 h post-seeding, for another 2 h. A cell viability curve was generated according to the 450 nM absorbance that was detected using a microplate reader (Bio-Rad, USA).

### 2.7 Wound-healing assay

Cells containing various transfections were seeded in 24-well plates (2 × 10^4^ cells/well) and cultured overnight until they reached 90% confluence. Sterile pipette tips were used to scratch a wound on the cell surface, and the wound distance was quickly captured using a light microscope (Leica, Germany). After 24 h of cell culture in culture medium without serum, the distance of the wounds was captured again using a microscope. The distance was used to assess the cell migratory capacity.

### 2.8 Flow cytometry

Flow cytometry was performed to detect cell apoptosis using an Annexin V-FITC Apoptosis Detection Kit (Beyotime). Cells (1 × 10^6^) were collected and resuspended in 100 μL 1× binding buffer. Annexin V-FITC and PI (1:1 in volume) were added to the cells for 20 min in the dark. The rate of apoptosis was determined using a flow cytometer (Beckman Coulter, USA). The sum of the upper and lower right quadrants represents the apoptosis rate.

### 2.9 Western blot

A commercial RIPA lysis reagent (Catalog No: CW2333; Cwbio) was used to extract total proteins. After quantification using the BCA Protein Assay Kit (Catalog No: CW0014; Cwbio), 20 μg of the protein sample was added to 10% sodium dodecyl sulfate-polyacrylamide gel electrophoresis to separate the protein bands. The separated proteins were transferred to a polyvinylidene fluoride membrane and blocked for 2 h in 5% milk at room temperature. Subsequently, the membrane was incubated overnight at 4°C with the primary antibody and the secondary antibody at room temperature for 2 h. Finally, the protein signals were scanned using an ECL kit (Abcam, USA). All antibodies were obtained from Abcam, including anti-Bax (Catalog No: ab32503; 1/2,000), anti-Bcl-2 (Catalog No: ab32124; 1/2,000), anti-Wnt (Catalog No: ab63934; 1/1,000), anti-Gsk-3β (Catalog No: ab131356; 1/1,000), anti-phosphorylated Gsk-3β (anti-p-Gsk-3β; Catalog No: ab75814; 1/10,000), anti-β-catenin (Catalog No: ab68183; 1/1,000), anti-c-Myc (Catalog No: ab168727; 1/1,000), anti-GAPDH (Catalog No: ab9485; 1/2,500), and HRP-labeled goat anti-rabbit IgG (Catalog No: ab205718; 1/5,000).

### 2.10 Animal study

Nude mice (Balb/c; 6 weeks old; *n* = 10) used for the experiment were acquired from Vital River (Beijing, China). shRNA targeting circ_0000285 (sh-circ_0000285), circ_0000285 overexpression (OE-circ), sh-NC, and OE-circ lentiviral vectors were purchased from OBiO Technology Shanghai Corp., Ltd. (Shanghai, China). sh-circ_0000285 sequences were synthesized and inserted into the pLV-CMV-puro-U6-lentiviral vector. SK-N-BE cells were then infected with lentivirus-packaged shRNA targeting circ_0000285 (sh-circ_0000285) or OE-circ for stable circ_0000285 knockdown or overexpresion using 2 μg/mL puromycin. Next, SK-N-BE cells (2 × 10^6^ cells/100 μL) were hypodermically injected into nude mice (two groups; *n* = 5 per group) to induce tumor formation. During tumor development, the volume (length × width^2^ × 0.5) was measured weekly. After 5 weeks, all experimental mice were euthanized under anesthesia. Tumor tissues were collected and analyzed. The Animal Research Procedures Committee of the Affiliated Hospital of Guizhou Medical University authorized animal care and use.

### 2.11 Dual-luciferase reporter assay

The wild-type (WT, containing miR-582-3p binding sites) and mutant-type (MUT, containing scrambled miR-582-3p binding sites) sequence fragments of circ_0000285 were synthesized by GenePharma (China) and cloned into the pmirGLO vector to construct the WT and MUT reporter vectors of circ_0000285. The experimental cells were co-transfected with miR-582-3p (miR-NC as a control) and the WT or MUT vector circ_0000285 and incubated for 48 h. Luciferase activity in the cells was detected experimentally using a dual-luciferase reporter kit (Catalog No: RG027; Beyotime).

### 2.12 RNA binding-protein immunoprecipitation (RIP) assay

Following the guidelines of the Magna RIP RNA-Binding Protein Immunoprecipitation Kit (Catalog No: 17-700; Millipore, USA), RIP experiments were performed to determine whether circ_0000285 was involved in miR-582-3p-governed RNA-induced silencing complex (RISC). Simply put, magnetic beads prepared with anti-IgG or anti-Ago2 coatings were exposed to cell lysates to capture RNA complexes linked to Ago2. RNA complexes were eluted from the beads and extracted for RT-qPCR.

### 2.13 Statistical analysis

Experimental data were collected from three independent biological experiments and then processed using GraphPad Prism 7.0 (GraphPad, USA). Data are presented as the mean ± standard deviation. Spearman’s correlation coefficients were calculated to evaluate linear correlations between the two groups. Student’s *t*-test or analysis of variance was used to compare differences. *P* values <0.05 indicate a significant difference.


**Ethics approval:** The study was approved by the research ethics committee of the Affiliated Hospital of Guizhou Medical University, Guizhou, China. The treatment of samples of clinical tissues is strictly in compliance with the Helsinki Declaration of Ethics. All patients who participate in the study have signed a written informed consent. This animal experiment was conducted in accordance with the ARRIVE guidelines. This animal experiment was authorized by the Animal Care and Use Committee of the Affiliated Hospital of Guizhou Medical University.
**Consent to participate:** All patients have signed an informed written consent.
**Consent for publication:** All participants gave their consent to publish.

## 3 Results

### 3.1 circ_0000285 showed a high expression level in NB tissues and cells

**Figure 1:**
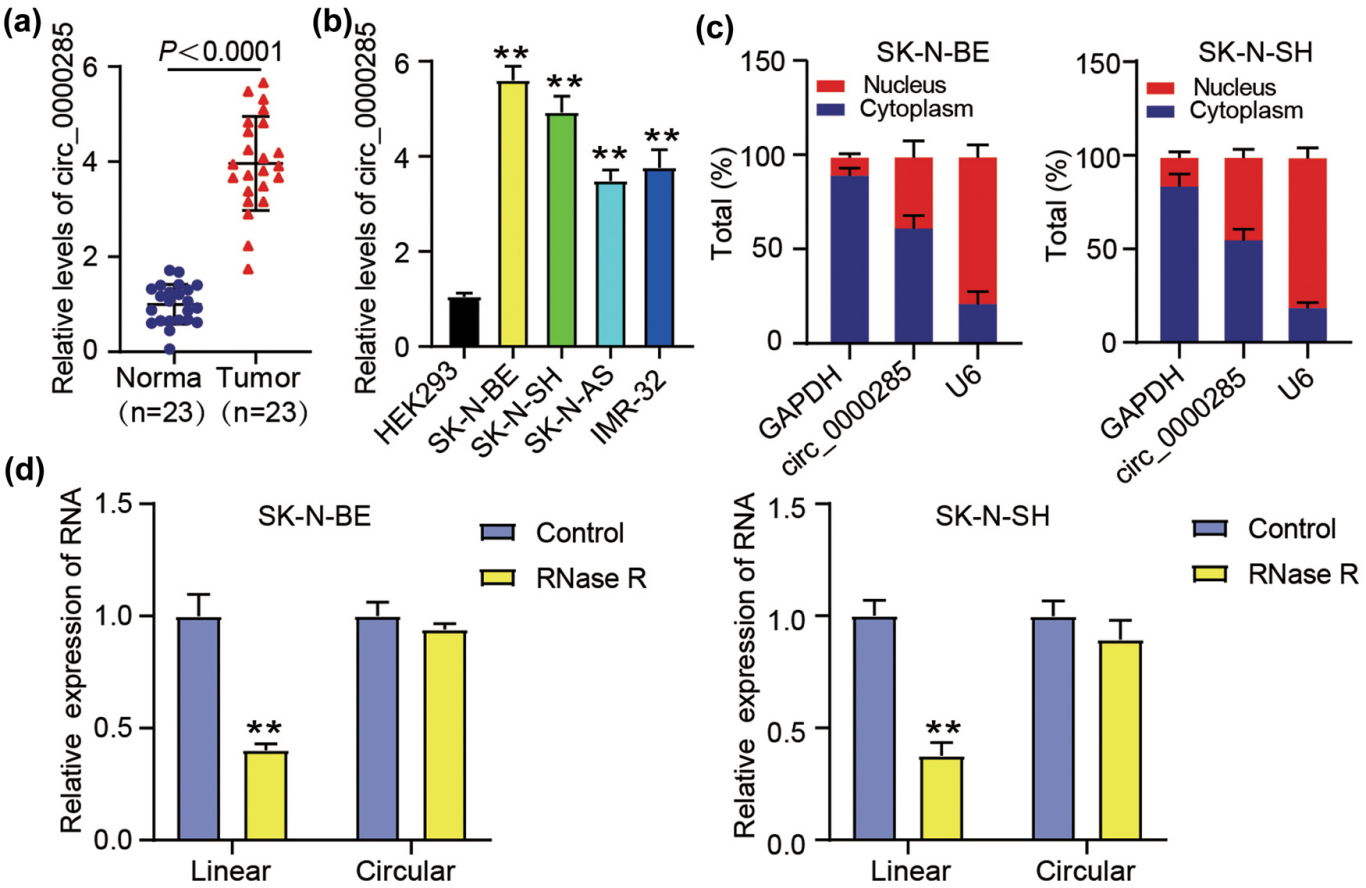
circ_0000285 showed a high expression level in NB tissues and cells. (a) The relative circ_0000285 expression in the NB and normal samples. (b) The relative circ_0000285 expression in non-cancer control cells (HEK293) and NB cells (SK-N-BE, SK-N-AS, SK-N-SH, and IMR-32), **P < 0.01 vs HEK293. (c) Subcellular distribution of circ_0000285 in nucleus or cytoplasm of SK-N-BE and SK-N-SH cells was ensured by RT-qPCR. (d) The circular structure of circ_0000285 was checked using RNase R, **P < 0.01 vs control.

First, we elucidated the expression pattern of circ_0000285 in NB cells. As shown in Figure 1a, circ_0000285 expression was higher in NB tumor tissues than in normal samples. After dividing the circ_0000285 high and low expression groups based on the mean circ_0000285 expression, circ_0000285 with high expression suggested a higher International Neuroblastoma Staging System stage (*P* = 0.0430) and MYCN amplification (*P* = 0.0361, Table 2). Meanwhile, circ_0000285 expression was also increased in several NB cell lines, including SK-N-BE, SK-N-SH, SK-N-AS, and IMR-32, compared to that in HEK293 cells (Figure 1b). The following examinations selected cells of SK-N-BE and SK-N-SH with a relatively high circ_0000285 expression. In addition, we found that circ_0000285 was mostly expressed in the cytoplasmic fraction of NB cells but not in the nuclear fraction (Figure 1c). Next, total RNA was treated with RNase R, and RT-qPCR data showed that the linear parental gene HIPK3 was prominently digested by RNase R, whereas circ_0000285 was rarely digested by RNase R (Figure 1d), verifying the circular structure of circ_0000285. These results suggest that dysregulation of circ_0000285 may be associated with NB development.

**Table 2:** The correlation between circ_0000285 expression and characteristics

Characteristics	All patients (*n* = 23)	circ_0000285 expression		*P*-value
		High	Low	
**Age (months)**				>0.9999
<10	13	6	7	
≥10	10	4	6	
**Gender**				>0.9999
Boys	11	5	6	
Girls	12	5	7	
**INSS stage**				0.0430
1–2	8	1	7	
3–4	13	7	6	
4s	2	2	0	
**MYCN amplification**				0.0361
Yes	12	8	4	
No	11	2	9	

INSS, International Neuroblastoma Staging System. *P*-value was analyzed by Fisher’s exact test.

### 3.2 circ_0000285 silencing decelerated NB cell growth and inactivated the Wnt/β-catenin signaling

**Figure 2:**
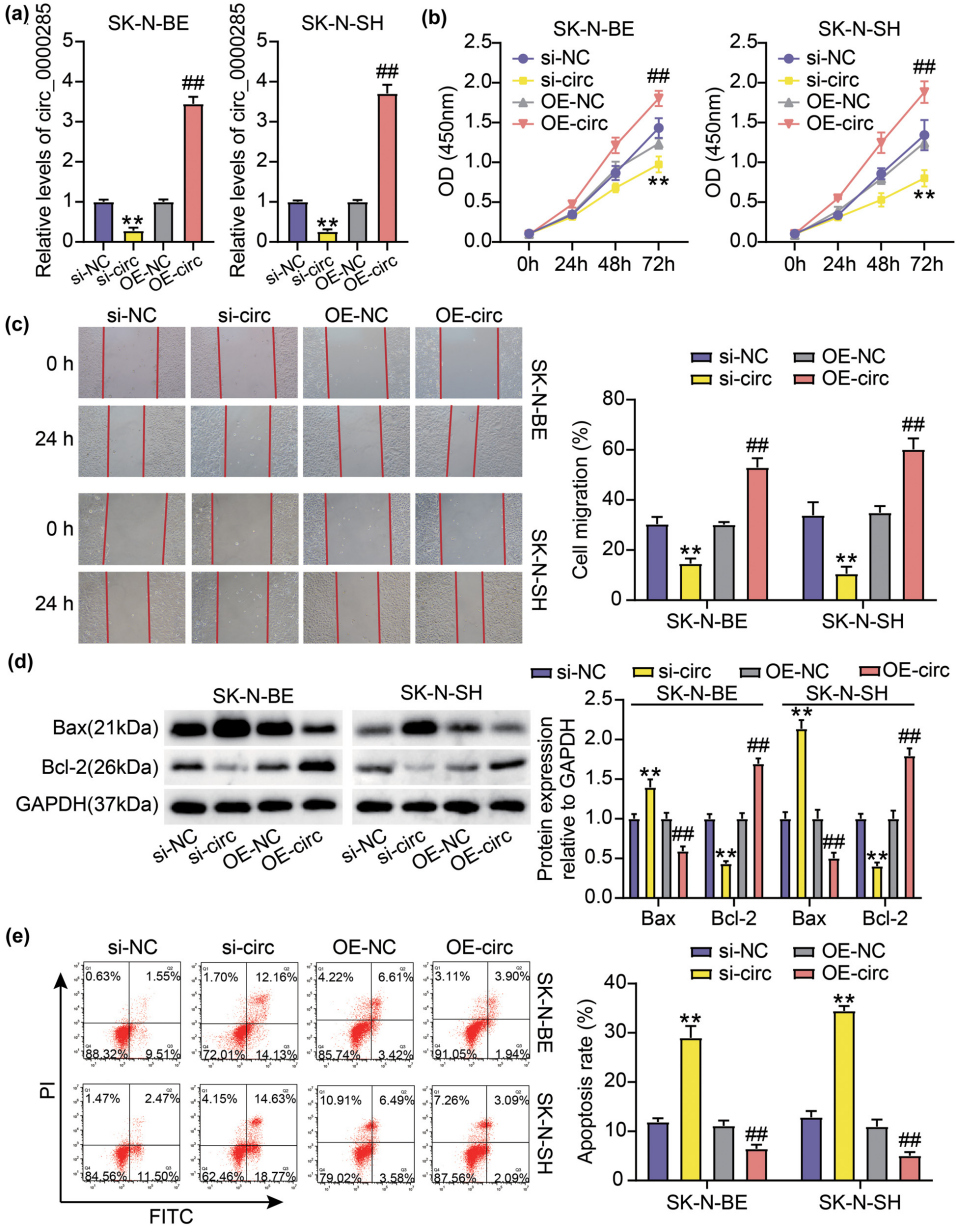
circ_0000285 silencing restrained NB cell growth *in vitro*. (a) circ_0000285 expression in si-circ, OE-circ, OE-NC, or si-NC-transfected SK-N-BE and SK-N-SH cells. (b) Cell proliferation in SK-N-SH and SK-N-BE cells after circ_0000285 downregulation or upregulation was evaluated by CCK-8 assay. (c) Cell migration in SK-N-BE and SK-N-SH cells after circ_0000285 absence or presence was detected by wound-healing assay. (d) The protein levels of Bax and Bcl-2 in SK-N-BE and SK-N-SH cells after circ_0000285 absence or presence were ascertained by western blotting. (e) The apoptosis rate in SK-N-BE and SK-N-SH cells after circ_0000285 absence or presence was detected by flow cytometry. **P < 0.01 vs si-NC; ^##^ *P* < 0.01 vs OE-NC.

**Figure 3:**
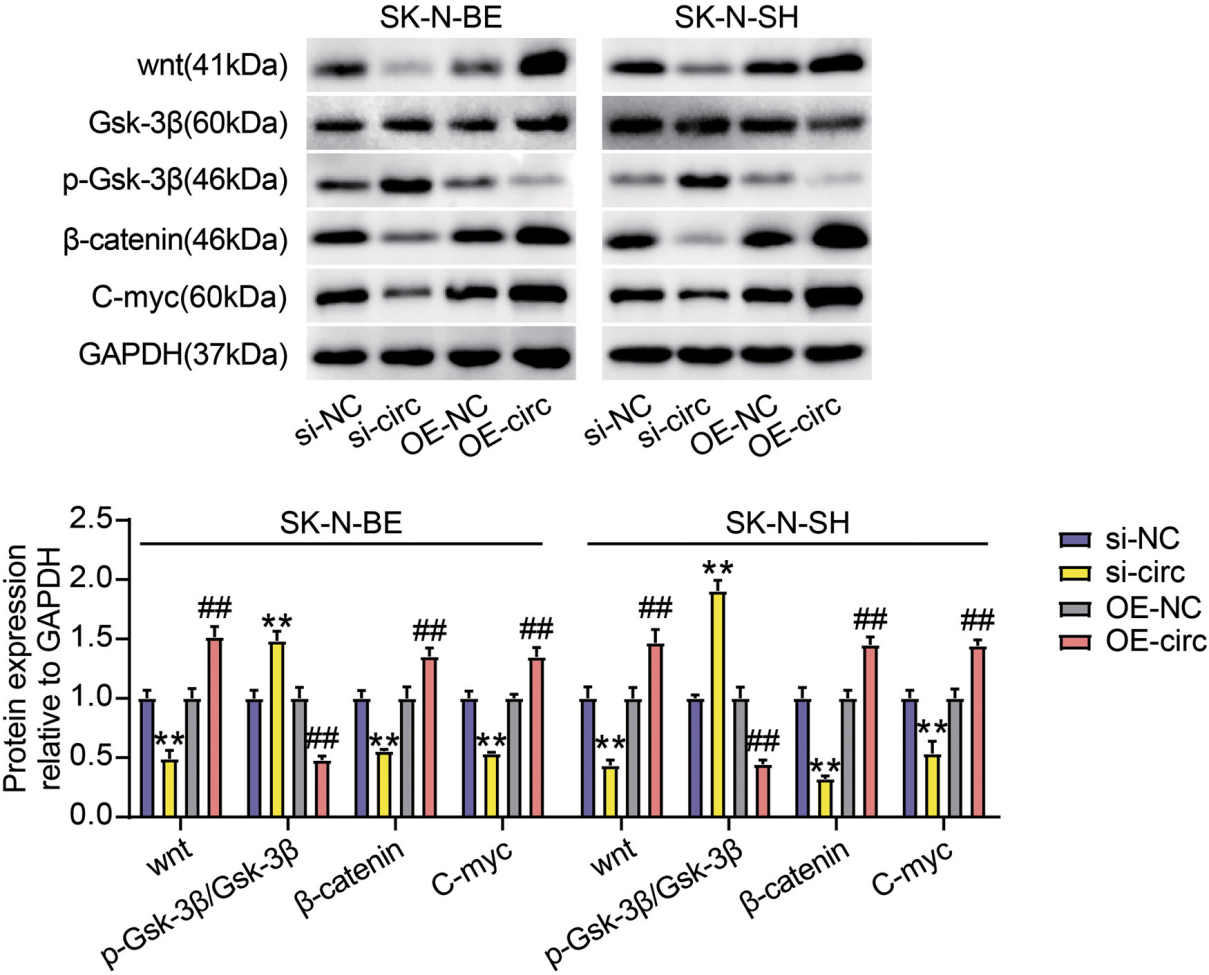
circ_0000285 silencing impaired the activity of Wnt/β-catenin signaling. The levels of wnt, Gsk-3β, β-catenin p-Gsk-3β, and C-myc proteins were measured by Western Blotting. **P < 0.01 vs si-NC; ^##^ *P* < 0.01 vs OE-NC.

To mediate circ_0000285 silencing and overexpression, si-circ and OE-circ were transfected into the SK-N-BE and SK-N-SH cells. We observed that circ_0000285 expression in experimental cells was considerably reduced after si-circ transfection, whereas circ_0000285 expression was enhanced after OE-circ transfection (Figure 2a). Next, the functions of circ_0000285 were investigated in detail. CCK-8 results showed that NB cells harboring circ_0000285 downregulation had impaired proliferative capacity, but NB cells with circ_0000285 transfection enhanced proliferative capacity (Figure 2b). Using a wound-healing assay, we observed that the migratory ability of SK-N-SH and SK-N-BE cells was strongly inhibited by circ_0000285 silencing, and the migratory ability of SK-N-SH and SK-N-BE cells was strongly promoted by circ_0000285 overexpression (Figure 2c). Western blot analysis revealed that enhanced Bax expression and impaired Bcl-2 expression caused by circ_0000285 silencing in SK-N-BE and SK-N-SH cells suggested that circ_0000285 knockdown induced cancer cell apoptosis, but circ_0000285 overexpression showed the opposite effect (Figure 2d). Flow cytometry further showed that the circ_0000285 knockdown induced apoptosis, and circ_0000285 overexpression suppressed apoptosis (Figure 2e). Moreover, the decrease of Wnt, β-catenin, and c-Myc levels and the increase of phosphorylated Gsk-3β level were observed in si-circ-transfected cells, but the increase of Wnt, β-catenin, and c-Myc levels and the decrease of phosphorylated Gsk-3β level were observed in OE-circ-transfected cells (Figure 3). In summary, circ_0000285 knockdown inhibited NB cell development, which might be associated with the inhibition of Wnt/β-catenin signaling.

### 3.3 circ_0000285 depletion decelerated tumor growth in animal models

**Figure 4:**
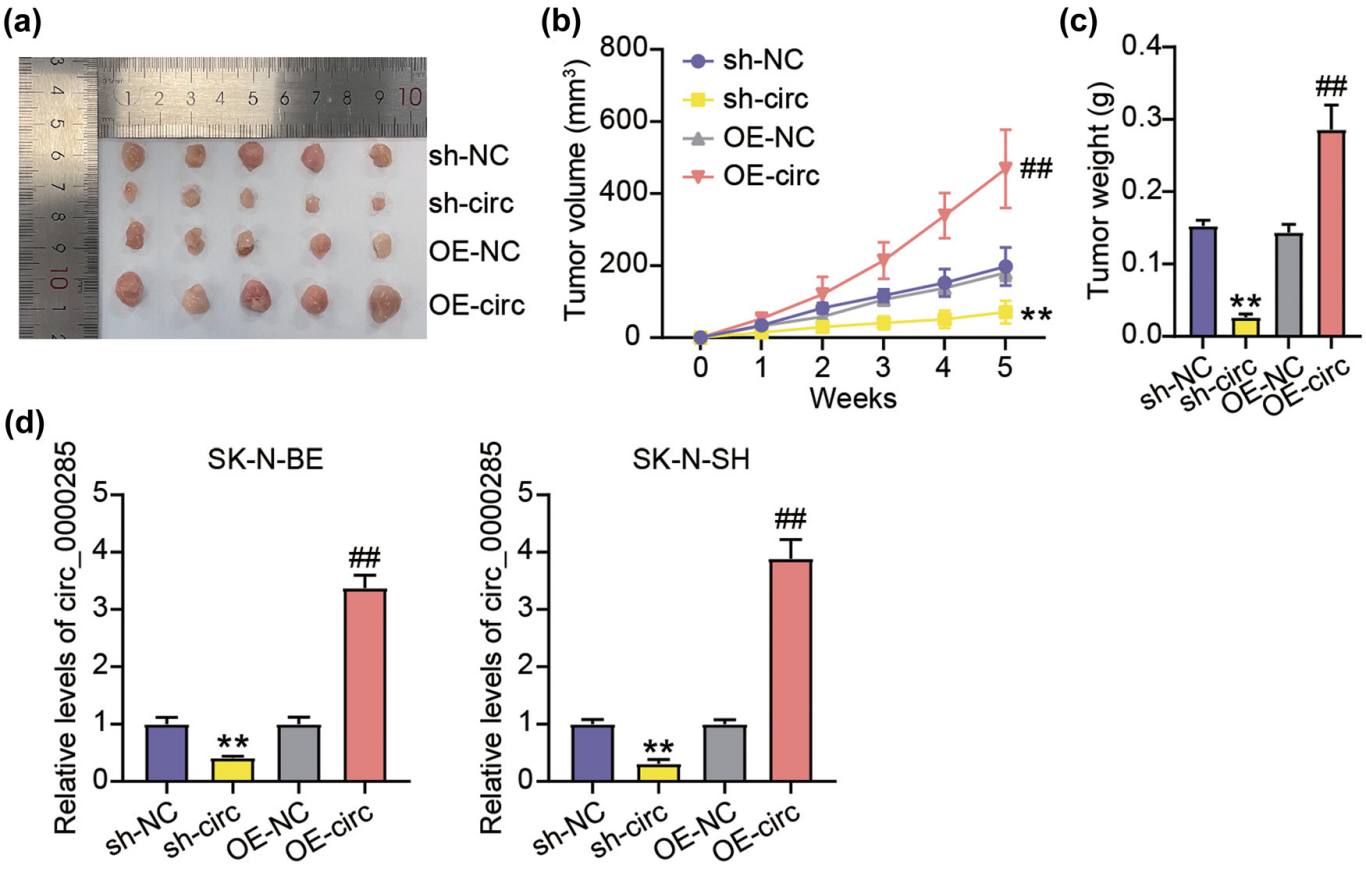
circ_0000285 silencing repressed tumor development in animal models. (a) The tumor images were taken. The volume (b) and weight of the tumor (c) in the tissue of the tumor in animal models were measured to evaluate the NB tumor growth *in vivo.* (d) The circ_0000285 expression in the tissues of the tumor in animal models was measured by RT-qPCR. **P < 0.01 vs sh-NC; ^##^ *P* < 0.01 vs OE-NC.

To induce tumorigenesis, SK-N-BE cells were infected with lentivirus particles of sh-circ or OE-circ to mediate stable circ_0000285 downregulation. As a result, we observed that tumor growth was dramatically decelerated in sh-circ-administered animals relative to sh-NC, whereas tumor growth was significantly enhanced in oe-circ-administered animals relative to OE-NC (Figure 4a–c). In tumor tissues removed from animals, sh-circ induced the decrease of circ_0000285 expression, and OE-circ induced the increase of circ_0000285 expression (Figure 4d). In summary, circ_0000285 depletion largely restrained NB tumorigenesis *in vivo*.

### 3.4 circ_0000285 targeted miR-582-3p whose expression was declined in NB

**Figure 5:**
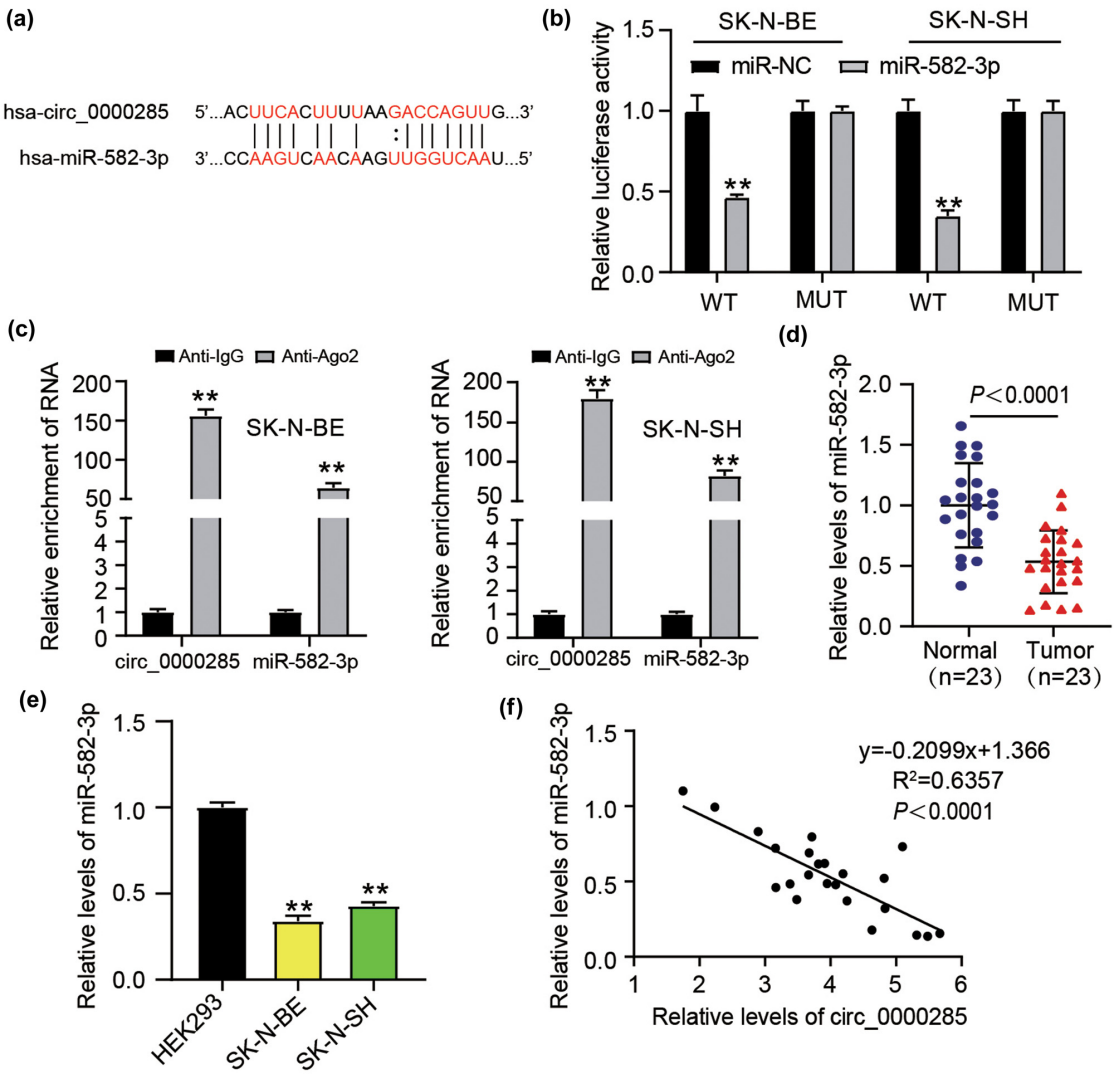
circ_0000285 targeted miR-582-3p. (a) The computational binding sites between miR-582-3p and circ_0000285 were acquired from Starbase (https://starbase.sysu.edu.cn/). (b) The putative binding sites between miR-582-3p and circ_0000285 were further verified by dual-luciferase reporter assay, **P < 0.01 vs miR-NC. (c) The binding of circ_0000285 to miR-582-3p was verified by RIP assay, **P < 0.01 vs Anti-IgG. (d) In NB and normal samples, relative miR-582-3p expression was found. (e) The relative miR-582-3p expression in non-cancer cells (HEK293) and NB cells (SK-N-BE and SK-N-SH), **P < 0.01 vs HEK293. (f) The correlation analysis of Spearman examined the association between the expression of miR-582-3p and the expression of circ_0000285 in NB samples.

In a canonical manner, we examined the downstream miRNAs targeted by circ_0000285 and clarified their functional mechanisms. Data from starBase showed that miR-582-3p is a hypothetical circ_0000285 target (Figure 5a). The binding sites between circ_0000285 and miR-582-3p, which were determined computationally, were then experimentally verified because deceased luciferase activity was found in the experimental cells containing the miR-582-3p and circ_0000285 wt constructs (Figure 5b). We then observed that a high abundance of circ_0000285 and miR-582-3p was enriched in anti-Ago2-mediated RIP, implying that circ_0000285 is involved in miR-582-3p-related RISC (Figure 5c). This evidence strongly validated the binding of circ_0000285 to miR-582-3p. Furthermore, miR-582-3p expression was significantly reduced in NB tumor tissues (Figure 5d) and cell lines (Figure 5e) relative to the matched non-cancerous samples. The expression of miR-582-3p and circ_0000285 were negatively correlated in NB tumor samples (Figure 5f). Taken together, miR-582-3p, a target of circ_0000285, showed an opposite expression pattern to that of circ_0000285 in NB.

### 3.5 circ_0000285 facilitated NB progression through Wnt/β-catenin pathway by targeting miR-582-3p

**Figure 6:**
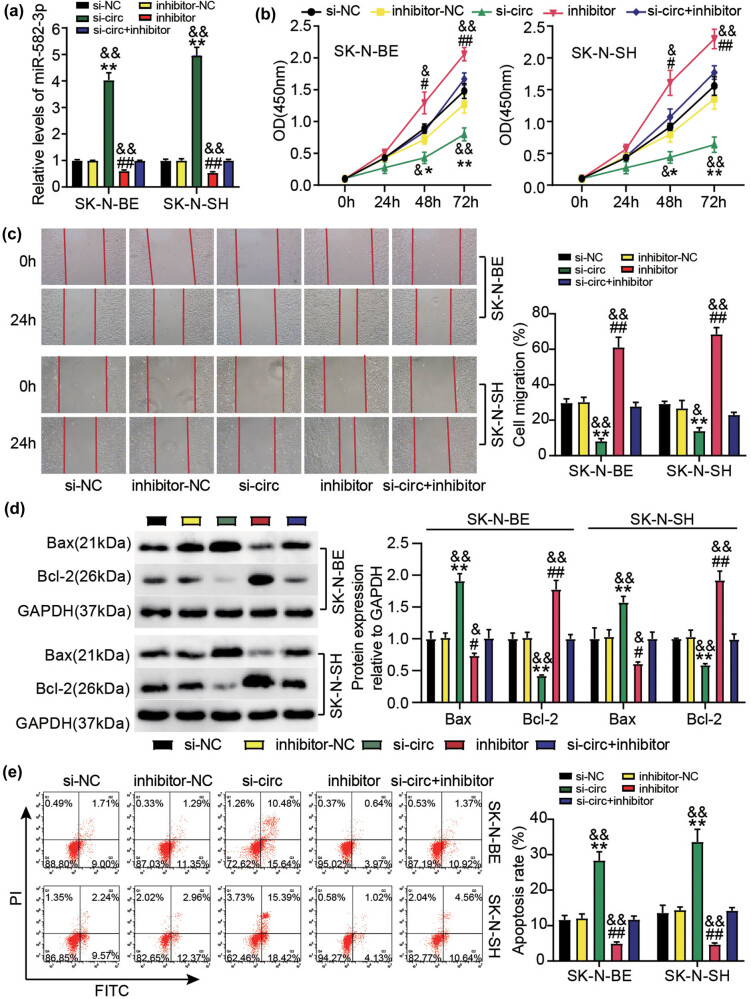
circ_0000285 knockdown inhibited NB cell functions via promoting miR-582-3p expression. (a–e) Following assays were investigated using SK-N-SH and SK-N-BE cells with si-circ, si-NC, inhibitor, inhibitor-NC or si-circ + inhibitor transfection. (a) The expression of miR-582-3p in these cells was ensured by RT-qPCR. (b) Cell proliferative ability in these cells was checked by CCK-8 assay. (c) Cell migration in these cells was checked by wound-healing assay. (d) In these cells, the expression of Bax and Bcl-2 both proteins was analyzed via Western blotting method. (e) Cell apoptosis in these cells was identified by flow cytometry. *P < 0.05, **P < 0.01 vs si-NC; ^#^ *P* < 0.01, ^##^ *P* < 0.01 vs inhibitor-NC; ^&^ *P* < 0.05, ^&&^ *P* < 0.01 vs si-circ + inhibitor.

**Figure 7:**
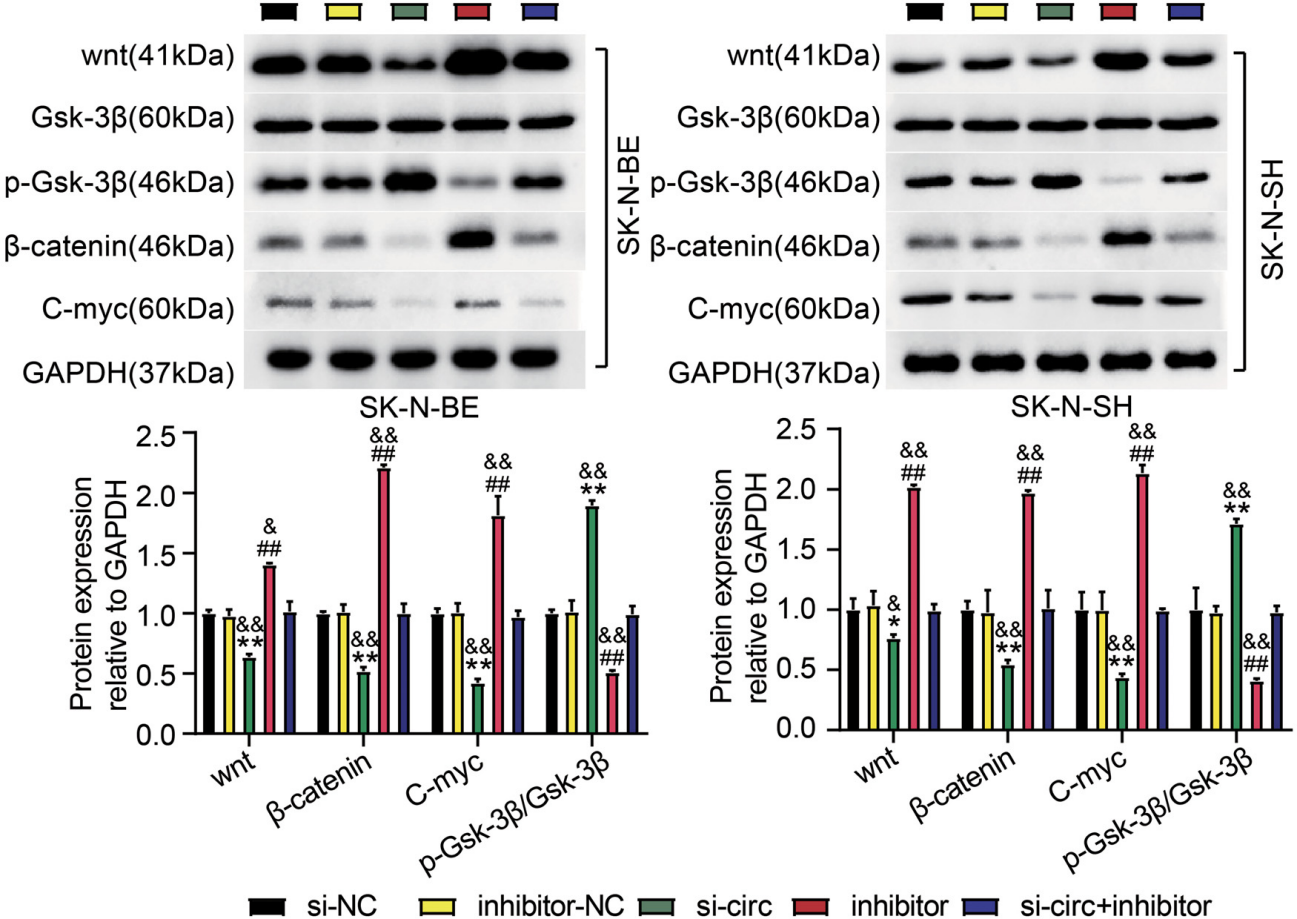
circ_0000285 knockdown inhibited NB cell functions through Wnt/β-catenin pathway. The protein levels of wnt, p-Gsk-3β, Gsk-3β, β-catenin and C-myc were determined by western blot. *P < 0.05, **P < 0.01 vs si-NC; ^##^ *P* < 0.01 vs inhibitor-NC; ^&^ *P* < 0.05, ^&&^ *P* < 0.01 vs si-circ + inhibitor.

To further explore the interactions between circ_0000285 and miR-582-3p, we transfected miR-582-3p inhibitors into si-circ-transfected NB cells and monitored functional changes. MiR-582-3p expression was significantly enhanced in si-circ cells, but was weak in inhibitor-transfected cells. MiR-582-3p expression was partially decreased by si-circ + inhibitor transfection relative to si-circ transfection in NB cells (Figure 6a). The functional study determined that cell proliferation of SK-N-BE and SK-N-SH was markedly aggravated by miR-582-3p inhibitor, and cell proliferative capacity suppressed by circ_0000285 silencing was considerably restored by additional miR-582-3p depletion (Figure 6b). Similarly, relative to the suppressive cell migration ability of NB cells after si-circ transfection, cell migration was substantially recovered in NB cells after si-circ + inhibitor transfection (Figure 6c). In addition, the circ_0000285 silencing-strengthened Bax expression was notably weakened by miR-582-3p depletion, while circ_0000285 silencing-reduced Bcl-2 expression was notably reinforced by miR-582-3p depletion (Figure 6d). After detecting the apoptosis rate by flow cytometry, the increase in the apoptosis rate caused by si-circ was reduced by the miR-582-3p inhibitor (Figure 6e). Moreover, the transfection of miR-582-3p inhibitor affected the expression of Wnt/β-catenin signaling proteins, indicating that additional inhibition of miR-582-3p enhanced the Wnt, β-catenin, and c-Myc levels that were decreased by circ_0000285 absence, and diminished the level of phosphorylated Gsk-3β that was heightened by circ_0000285 absence (Figure 7). These data indicate that circ_0000285 modulates miR-582-3p expression to affect Wnt/β-catenin signaling activity, thus regulating NB cell functions.

## 4 Discussion

In addition to their abundant and stable expression, circRNAs have been found in exosomes, urine, saliva, and other body fluids in clinical practice, and circRNAs have attracted much attention as promising biomarkers and therapeutic targets for cancers. Therefore, the role of circRNAs in various cancers should be systematically investigated. Here, we characterized the function of circ_0000285, the expression of which was aberrantly upregulated in NB. Silencing of circ_0000285 repressed NB cell growth and tumorigenesis and depressed Wnt/β-catenin signaling activity, which was partially attributed to the regulation of circ_0000285 on miR-582-3p. These findings, for the first time, verify the cancer-promoting effects of circ_0000285 in NB.

Recently, several circRNAs have been functionally identified in NB. For instance, circKIF2A is expressed in NB tissues and cells at higher levels, and circKIF2A knockdown attenuates NB cell growth, migration, invasion, and glycolysis energy metabolism to block NB progression [18]. Similarly, circCUX1 was also highly expressed in NB and predicted poor prognosis, and circCUX1 repression restrained aerobic glycolysis and cell aggressiveness in NB cells [19]. Furthermore, the role of circ_0000285 in several cancers has been elucidated. Zhang et al. reported that circ_0000285 was strongly expressed in osteosarcoma cell lines, and ectopic circ_0000285 expression aggravated the proliferative and migratory potencies of osteosarcoma cells [12,20]. A high abundance of circ_0000285 was also identified in cervical cancer, and circ_0000285 downregulation attested to the cancer cell cycle and provoked cell apoptosis and autophagy to repress cervical cancer development [13]. Consistent with these findings, we exhibited elevated circ_0000285 expression in NB tissue samples and cells for the first time. In *in vitro* analysis, we found that circ_0000285 depletion repressed NB cell proliferation and migration while provoking apoptosis. For *in vivo* analysis, we found that the absence of circ_0000285 blocked NB tumorigenesis in nude mice. These results confirm the carcinogenic effects of circ_0000285 on NB development.

Based on the starBase prediction, we found that miR-582-3p is a target miRNA of circ_0000285. Due to the limited research on the miR-582-3p function in NB, we chose miR-582-3p for analysis. A dual role of miR-582-3p has been reported in various cancers. miR-582-3p is poorly expressed in metastatic prostate cancer (PCa) tissues, and miR-582-3p enrichment represses PCa cell migratory and invasive abilities [16]. In addition, miR-582-3p expression in gastric cancer is low, and its accumulation of miR-582-3p considerably restricted the invasion, migration, and proliferation capabilities of gastric cancer cells [17]. miR-582-3p, on the other hand, is highly expressed in lung cancer and cervical adenocarcinoma, and forced miR-582-3p expression contributes to cancer cell migration, invasion, and survival [21,22]. Our results identified the downregulation of miR-582-3p in NB specimens and cell lines and discovered that miR-582-3p deficiency aggravated NB cell proliferative/migratory capacities but repressed apoptosis. Importantly, miR-582-3p deficiency, at least in part, reversed the functional effects of circ_0000285 knockdown and restored the malignant functions of NB cells.

Accumulating research has established that the activation of the Wnt/β-catenin pathway is related to aggressive cancer stem cell renewal, cell proliferation, differentiation, and tumorigenesis, and targeted Wnt/β-catenin pathway inhibition has been proposed as a new strategy against numerous malignant cancers [23,24,25]. Studies have shown that the activation of Wnt/β-catenin signaling is inhibited by the binding of Wnt ligands to frizzled/lipoprotein receptor-related protein coreceptors, which triggers Ser9 phosphorylation of Gsk-3β, leading to Gsk-3β inactivation and subsequent β-catenin accumulation and nuclear translocation [26,27,28]. After reviewing the literature, we found that miR-582-3p regulates the Wnt/β-catenin signaling pathway to participate in the progression of lung cancer and hepatocellular carcinoma [29,30]. However, whether miR-582-3p regulates the Wnt/β-catenin signaling pathway to regulate NB progression remains unknown. Therefore, we selected the Wnt/β-catenin signaling pathway. In our study, we discovered that decreased expression of Wnt, β-catenin, and c-Myc and enhanced expression of phosphorylated Gsk-3β were observed in NB cells after circ_0000285 knockdown, suggesting that circ_0000285 knockdown inactivated the Wnt/β-catenin signaling pathway. Moreover, circ_0000285 knockdown-weakened the Wnt/β-catenin signaling pathway was promoted by additional miR-582-3p deficiency, indicating that circ_0000285 knockdown enhanced miR-582-3p expression, thus blocking NB progression, which might be implicated in the inactivation of the Wnt/β-catenin signaling pathway.

Although the current study partly addressed the functional role and mechanism of circ_0000285 in NB, more detailed functions of circ_0000285 should be explored in future studies. In addition, the downstream functional genes of the circ_0000285/miR-582-3p pathway were not characterized in our current study and need to be explored to further understand the regulatory networks of circ_0000285. The correlation between circ_0000285 expression and clinicopathological characteristics of patients with NB is not well documented and requires further investigation.

## 5 Conclusion

Our study identified high expression of circ_0000285 in NB. circ_0000285 could function as a miR-582-3p sponge to activate Wnt/β-catenin signaling and thus facilitate the malignant progression of NB. circ_0000285, an oncogenic factor, may be a potential therapeutic target for NB.
